# The Photoinitiated Reaction Pathway of Full-length Cyanobacteriochrome Tlr0924 Monitored Over 12 Orders of Magnitude*

**DOI:** 10.1074/jbc.M114.566133

**Published:** 2014-05-09

**Authors:** Anna F. E. Hauck, Samantha J. O. Hardman, Roger J. Kutta, Gregory M. Greetham, Derren J. Heyes, Nigel S. Scrutton

**Affiliations:** From the ‡ Manchester Institute of Biotechnology and Photon Science Institute, Faculty of Life Sciences, The University of Manchester, Manchester M13 9PL, United Kingdom and; the § Central Laser Facility, Research Complex at Harwell, Science and Technology Facilities Council, Harwell Oxford, Didcot OX11 0QX, United Kingdom

**Keywords:** Biophysics, Cyanobacteria, Photobiology, Spectroscopy, Ultraviolet-visible Spectroscopy (UV-visible Spectroscopy)

## Abstract

The coupling of photochemistry to protein chemical and structural change is crucial to biological light-activated signaling mechanisms. This is typified by cyanobacteriochromes (CBCRs), members of the phytochrome superfamily of photoreceptors that exhibit a high degree of spectral diversity, collectively spanning the entire visible spectrum. CBCRs utilize a basic *E*/*Z* isomerization of the bilin chromophore as the primary step in their photocycle, which consists of reversible photoconversion between two photostates. Despite intense interest in these photoreceptors as signal transduction modules a complete description of light-activated chemical and structural changes has not been reported. The CBCR Tlr0924 contains both phycocyanobilin and phycoviolobilin chromophores, and these two species photoisomerize in parallel via spectrally and kinetically equivalent intermediates before the second step of the photoreaction where the reaction pathways diverge, the loss of a thioether linkage to a conserved cysteine residue occurs, and the phycocyanobilin reaction terminates in a red-absorbing state, whereas the phycoviolobilin reaction proceeds more rapidly to a final green-absorbing state. Here time-resolved visible transient absorption spectroscopy (femtosecond to second) has been used, in conjunction with time-resolved IR spectroscopy (femtosecond to nanosecond) and cryotrapping techniques, to follow the entire photoconversion of the blue-absorbing states to the green- and red-absorbing states of the full-length form of Tlr0924 CBCR. Our analysis shows that Tlr0924 undergoes an unprecedented long photoreaction that spans from picoseconds to seconds. We show that the thermally driven, long timescale changes are less complex than those reported for the red/far-red photocycles of the related phytochrome photoreceptors.

## Teaser

**Background:** Cyanobacteriochromes (CBCRs) are spectrally varied photoreceptors with diverse photoconversion mechanisms.

**Results:** The photoinitiated conversion of CBCR Tlr0924 between states appears to proceed in two steps covering timescales from femtoseconds to seconds.

**Conclusion:** The evidence presented suggests a less complex mechanism than in related photoreceptors.

**Significance:** Knowledge of the full CBCR photoconversion is critical to future exploitation of the photoreceptor family.

## Introduction

**FIGURE 1.**
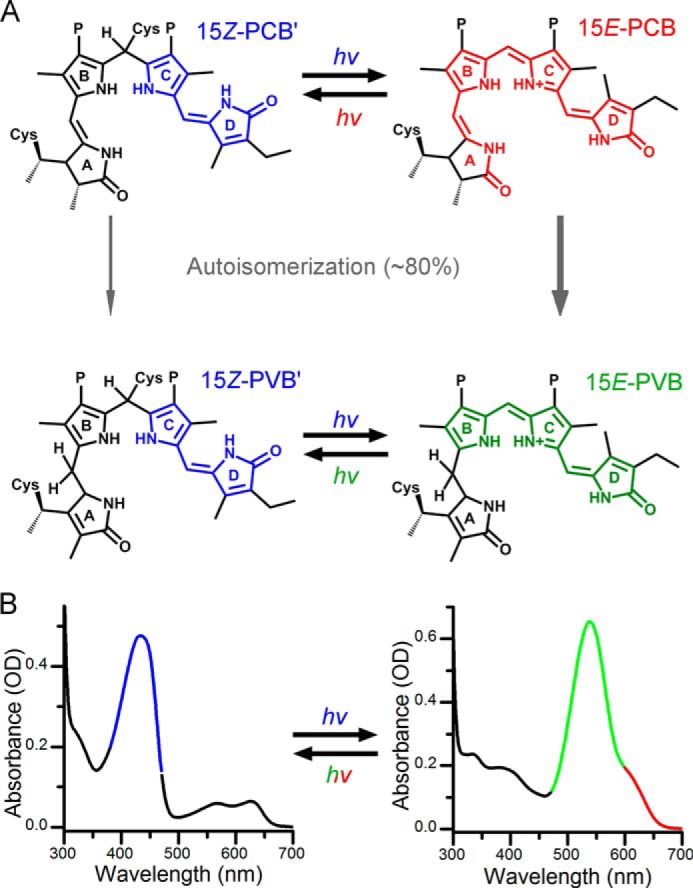
**Structures (*A*) and related absorption spectra (*B*) of the ^15^*^Z^*^-PVB′^Pb, ^15^*^E^*^-PVB^Pg, ^15^*^Z^*^-PCB′^Pb, and ^15^*^E^*^-PCB^Pr chromophores of Tlr0924.** The PCB and PVB populations autoisomerize to yield a mixture of the two chromophores, which can be converted between photostates with the relevant wavelength of light.

Photon-driven *E*/*Z* isomerization is the elementary chemical reaction that initiates the biological function of a large group of photoreceptors (1). Phytochromes and cyanobacteriochromes (CBCRs)^3^ form part of this family and mediate vital photomorphogenic processes in plants and photoadaptive behavior in microorganisms (2, 3). The two families share the very basic unit of light sensing: an open chain bilin chromophore covalently anchored via a thioether linkage to the protein, which is nestled in a GAF (cGMP-specific phosphodiesterase/adenylyl cyclase/FhlA protein) domain. The bilin can exist in two different states, which are governed by a reversible photon-driven *E*/*Z* isomerization of the methine bridge between rings C and D (Fig. 1*A*). This photocycle has been studied in detail in the structurally and spectrally conserved phytochromes (1, 3–6). The exclusively red/far-red conversion of phytochromes involves an ultrafast photoisomerization of the bilin cofactor followed by a number of slower, thermally driven, conformational changes to trigger the signaling response (4). Photoactivity depends on the presence of flanking PAS (Per, Arnt, Sim) and PHY (phytochrome-specific) domains (6). In contrast, CBCR GAF domains autonomously undergo efficient, reversible, photoconversions in which different modifications to their covalently bound chromophore allow sensitivity to the entire UV-visible spectrum (5, 7). Modulation of the bilin-conjugated system is achieved by different tuning mechanisms, such as structural variations in the chromophore and/or breakup of the delocalized π-electron system. However, the photochemical mechanisms that allow this photosensory flexibility are only beginning to be understood, and a kinetic description of the complete photocycle of a CBCR is still lacking.

Tlr0924 from *Thermosynechococcus elongatus* is a blue/green photoreceptor that belongs to the D*X*CF subgroup (containing the Asp-Xaa-Cys-Phe motif) of CBCRs (5). In a process that has been observed previously in similar CBCRs (8, 9) the intrinsic GAF isomerase activity in Tlr0924 converts the phycocyanobilin (PCB) precursor chromophore to phycoviolobilin (PVB) incompletely on a timescale of days, resulting in a heterogeneous population with a final ratio of approximately 1:4 (Fig. 1*A*). The absorption spectrum is consequently composed of features originating from both the PVB and PCB chromophores (Fig. 1*B*). The Tlr0924 photocycle has been studied by visible absorption spectroscopy and circular dichroism and was shown to interconvert between blue-absorbing (Pb) and green-absorbing (Pg) states upon absorption of the relevant wavelength of light (5, 10). The large spectral shift cannot be achieved by isomerization alone, but is brought about by a second thioether linkage formed between the bilin C10 and Cys-499 in the protein DXCF motif (10–12). When the PVB and PCB chromophores are linked to the protein they become rubin-like structures with a saturated C10, referred to here as PVB′ and PCB′, respectively. The absorption maxima of the Pb ground states are spectrally identical for PCB′ and PVB′ in the visible region because the second thioether linkage restricts the photochemically relevant conjugated system to the C and D rings. Blue light absorption will therefore inevitably activate both photoconversions, resulting in green-absorbing PVB photoproduct and red-absorbing (Pr) PCB photoproduct, with their conjugated systems extended to ring B and rings A and B, respectively. The photoproducts still exhibit a significant spectral overlap, and green light absorption returns both chromophores to their blue-absorbing states. However, far-red illumination will convert only PCB back to the Pb state. The individual spectral contributions can be isolated by this sequential photoconversion permitting further studies (8, 11), or the states without the second thioether linkage to the protein can be visualized by (permanent) acid denaturation (8, 13, 14).

The ultrafast photoisomerization reaction dynamics of the GAF domain of Tlr0924 have previously been investigated by exciting the two parallel photoconversions simultaneously, which has allowed the primary photoproducts to be identified (15). In the present work we have used a combination of time-resolved spectroscopies covering femtosecond through to second timescales in the visible spectral region, femtosecond to nanosecond timescales in the IR spectral region, and cryotrapping techniques to characterize all steps in the forward photoreaction (Pb to Pg and Pr) of the full-length protein. The experimental isolation of the PVB and PCB populations allowed a detailed assignment of the intermediates formed. It is shown that the primary photoproducts are converted to the final Pg and Pr states by the removal of the second thioether linkage in a single, thermally driven step on the millisecond to second timescale. This detailed kinetic and spectroscopic characterization demonstrates that the photoconversions of CBCRs are significantly different from the highly complex photocycles found in the related phytochrome proteins (16–20).

## EXPERIMENTAL PROCEDURES

### 

#### 

##### Protein Expression and Purification

The gene for full-length Tlr0924 was synthesized (Genscript Inc.) and cloned into the pBAD-HisB expression plasmid. Full-length Tlr0924 was expressed as a recombinant holoprotein by using a dual plasmid *Escherichia coli* expression system (4, 21). BL21(DE3) containing a PCB biosynthetic expression plasmid (pCOLADuet-1 (Novagen) HO1 PcyA) for chromophore production was cotransformed with the pBAD-HisB-Tlr0924 plasmid. The protein was purified by a two-step method employing nickel affinity chromatography followed by gel filtration in a phosphate-based buffer system (100 mm sodium-potassium phosphate, 300 mm NaCl, pH 7) supplemented with 200 mm l-histidine for elution. Pure sample was flash-frozen and stored at −80 °C. The chromophore content was ∼80% PVB and 20% PCB, as indicated by UV-visible spectroscopy, and based on published conversion rates this ratio can be assumed to remain constant over the course of the experiments (5).

##### Ultrafast Transient Absorption Spectroscopy

**FIGURE 2.**
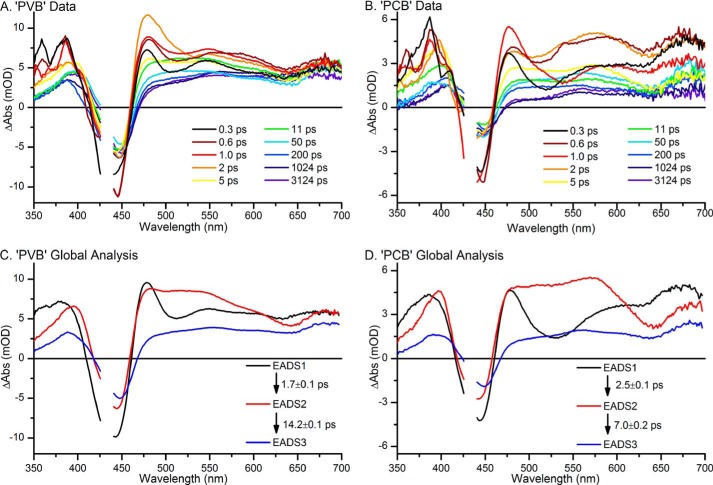
*A* and *B*, ultrafast transient absorption spectra, collected after excitation at 435 nm, at selected time points for “PVB” samples (*A*), where Pb states were constantly regenerated with green light, and “PCB” samples (*B*), where the Pb states were constantly regenerated with red light. *C* and *D*, global analysis of the ultrafast transient absorption data for the “PVB” (*C*) and “PCB” (*D*) samples yielded three EADS that sequentially interconvert.

The laser system used in the visible transient absorption measurements consists of a Ti:sapphire amplifier (hybrid Coherent Legend Elite-F-HE) pumped by a Q-switched Nd:YLF laser (Positive light Evolution-30) and seeded by a Ti:sapphire laser (Spectra Physics Mai Tai). The amplifier output (1-kHz repetition rate, 800-nm center wavelength, ∼120-fs pulse duration) was split to generate the pump and probe beams. A noncollinear optical parametric amplifier (Light Conversion TOPAS White) was used to generate the pump beam centered at 435 nm, with a full width at half-maximum intensity of approximately 10 nm. Excitation energies of 0.75–1 mJ were used with a beam diameter of approximately 150 μm, which yielded pump fluences of 4.2–5.7 mJ/cm^2^. The accessible region of the spectrum was maximized by adjusting the polarizations of pump and probe to be perpendicular and using a polarizer before the detectors to eliminate a large proportion of the scattered pump light. Data collected with a depolarized pump beam yielded kinetics and spectra similar to those shown in Fig. 2, although the intensity of the negative stimulated emission peak at ∼510 nm varied between the samples, thus we assume any polarization effects will not affect the model derived from these data. The probe beam consisted of a white light continuum generated in a rastered CaF_2_ crystal. The broad band pump-probe transient absorbance spectrometer Helios (Ultrafast Systems LLC) had a time resolution of ∼0.2 ps. Absorbance changes were monitored between 350 and 700 nm with data points collected randomly over the 3-ns time frame. Samples were contained in stirred 2-mm path length quartz cuvettes (optical density at 535 nm = 0.5). During the measurements the samples were continuously illuminated using a cold light source (Schott KL1500), and the appropriate bandpass filter (Andover Corp). Illumination at 540 nm was used to regenerate the PVB′ and PCB′ Pb states from their corresponding PVB Pg and PCB Pr states, and 640-nm illumination was used to regenerate the PCB′ Pb state from the PCB Pr state.

Time-resolved IR spectroscopy was carried out at the Ultra facility (CLF, STFC Rutherford Appleton Laboratory, UK), which uses a 10-kHz repetition rate laser and has a time resolution of approximately 100 fs (22). Samples were flowed through a 100-μm CaF_2_ measurement cell and the sample holder rastered to avoid sample damage. In addition, the Pb states of the sample were regenerated by continuous sample illumination with a cold light source as described above. An excitation energy of 0.6 μJ at 435 nm was used, the beam diameter was approximately 150 μm, yielding a pump fluence of 4.3 mJ/cm^2^, and the excitation beam was set at the magic angle with respect to the IR probe beam. Data were collected for approximately half an hour per dataset, the spectral resolution was ∼3 cm^−1^, pixel to wavenumber calibration was performed as described previously (23).

##### Laser Flash Photolysis

A Q-switched Nd:YAG laser (Brilliant B, Quantel) was used for sample excitation in single shot mode. 435-nm pump pulses were generated via an optical parametric oscillator, the pulse duration was 6–8 ns, and energies of 5–30 mJ were used (depending on the set of measurements). The beam diameter was on the order of 1 cm, yielding pump fluences of 6.4–38 mJ/cm^2^. The detection system (Applied Photophysics Ltd.) consisted of a 150-W xenon arc lamp and monochromators on either side of the sample holder, placed at right angles to the incident pump beam. Measurements on the millisecond to second scale were recorded using a photomultiplier tube. For shorter timescale measurements the probe beam was pulsed and kinetic traces recorded on a digital oscilloscope (Agilent Technologies, Infiniium, 54830B). The 300–700-nm region was monitored by recording absorption transients in 5-nm steps, each data point being the average of three or more transients. The appropriate photostate was regenerated after each shot by illumination with a cold source lamp fitted with the relevant bandpass filters as described above. The sample was contained in a 1-cm path length quartz cuvette and maintained at 25 °C by a circulating water bath. The sample had an OD of 0.2–0.8 at the excitation wavelength depending on the species excited, samples were frequently replaced, and their quality monitored by UV-visible Spectroscopy (Varian, Cary 50).

##### LED Flash Photolysis

Analogous to the laser flash experiment, which was used to cover the nanosecond to millisecond dynamics of Tlr0924, a commercial UV-visible spectrometer (Varian, Cary 50) was used to cover the dynamics in the millisecond to minute dynamics of PCB in Tlr0924. The sample was excited orthogonally by a pulsed high power LED (M455L3, Thorlabs) with a flash of 10-ms duration and 10-mJ pulse energy, which was collimated by an argon-coated aspheric lens (Thorlabs) to yield a pump fluence of approximately 5 mJ/cm^2^. A small sample volume of 0.5 ml with an OD of ∼0.2 at the excitation wavelength was used, allowing excitation of the entire sample volume to avert diffusion effects on the long timescales measured. The absorbance changes were recorded at single wavelengths ranging from 305 to 675 nm in 10-nm steps with a time resolution of 12.5 ms in a time window of 30 s. Illumination at 640 nm was used to regenerate the PCB Pb state from the Pr state between datasets. The LED flash was manually triggered for each time trace and all datasets corrected to the same time zero during data processing. The raw data, consisting of 2400 data points, were reduced to 500 points by averaging according to a logarithmic time axis prior to analysis.

##### Cryotrapping

Samples were prepared at an OD at 435 nm of ∼1 in cryogenic buffer (phosphate-based buffer system (100 mm sodium-potassium phosphate, 300 mm NaCl, pH 7) made using 22% water, 30% glycerol, and 48% sucrose) and cooled to 77 K in a cryochamber (Optistat DN liquid nitrogen cryostat, Oxford Instruments Inc.) in the desired state. The sample was then allowed to warm up in 10-K steps up to 327 K, illuminated at 430 nm for 10 min at each temperature point, and recooled to 77 K prior to recording a UV-visible absorbance spectrum (Cary 50, Agilent Technologies). Photoconversion was achieved by illumination with a cold light source as described above.

##### Global Analysis

The transient absorption and laser flash photolysis three-dimensional datasets were analyzed globally using the open-source software Glotaran (24). This procedure reduces the matrix of time, wavelength, and change in absorbance to one or more exponentially decaying time components, each with a corresponding difference spectrum. These spectral and lifetime components can then be used to identify individual photoproducts of the reactions. The data were fitted with a sequential, unbranched, unidirectional model, which shows the spectral evolution from one component to another, and yielded evolution-associated difference spectra (EADS). It was assumed that all states of Tlr0924, including the intermediates in the photoconversion processes, have Gaussian-shaped absorption profiles. Using this assumption the EADS were fitted with Gaussian functions to identify, and obtain accurate peak positions, of the individual intermediates. The visible and IR ultrafast transient absorption data were fitted from 0.3 and 0.2 ps, respectively, to avoid any contributions from coherent artifacts (25).

## RESULTS

### 

#### 

##### Ultrafast Transient UV-visible Dynamics

Previous measurements have shown that the photoisomerization of the bilin cofactor occurs on an ultrafast timescale for both phytochromes and the isolated GAF domain of CBCRs (15, 16, 19, 26–32). Here, ultrafast transient absorption spectroscopy has been used to study the *Z* to *E* photoisomerization process in the full-length Tlr0924 CBCR. Laser pulses at 435 nm were used to induce photoconversion of the ^15^*^Z^*^-PVB′^Pb and ^15^*^Z^*^-PCB′^Pb populations to the ^15^*^E^*^-PVB^Pg and ^15^*^E^*^-PCB^Pr populations, respectively. Continuous illumination with green light (540 nm) during data collection returned both of the “final” ^15^*^E^*^-PVB^Pg and ^15^*^E^*^-PCB^Pr populations to their corresponding Pb states. Therefore excitation at 435 nm initiated the photoisomerization of a mixture of both PCB′ and PVB′ Pb states. However, as Tlr0924 exists as a heterogeneous population of PVB and PCB in a ratio of ∼4:1 the majority of the time-dependent absorption changes are dominated by PVB (∼80% of the signal). Further separation of the species was made possible by illumination with red light (640 nm) during data collection, which converted only the ^15^*^E^*^-PCB^Pr population to the Pb state, leaving the PVB population in the Pg state. In this case, the majority of signal after excitation at 435 nm originated from PCB only.

Using the conditions defined above, ultrafast transient absorption difference spectra were measured for both the predominantly PVB (hereafter termed “PVB”) and the predominantly PCB (hereafter termed “PCB”) forward reactions (Fig. 2, *A* and *B*). In both cases, there is a bleach of the main ground state absorption band (GSB) at ∼436 nm, which is flanked by broad overlapping positive excited state absorption (ESA) signals. We also observed a small ∼510-nm stimulated emission (SE) band at very early times, and a small negative feature at ∼640 nm, which likely corresponds to the bleach of a previously observed ground state absorption peak attributed to a small amount of inactive or modified protein (10). On the sub-ps to 3-ns timescale monitored in these experiments the magnitude of the GSB is reduced and appears to slightly red shift. The features in the red region of the spectrum disappear simultaneously and result in a broad positive feature spanning the entire 500–800-nm region at 3 ns. The data collected for the “PCB” sample show very similar spectral features to the “PVB” sample although the SE band at ∼510 nm is more pronounced for the “PCB” sample and appears to be slightly red-shifted. This may be due to contamination of the signal by a small amount of PVB Pg to Pb photoconversion, which would result in a bleach feature at 532 nm. The bilin structures of the two chromophores are broadly similar, with the conjugated systems being identical in PCB and PVB, hence photoexcitation is likely to result in correspondingly similar excited states and primary photoproducts.

The datasets were analyzed globally using a sequential model to give rise to EADS, which represent the spectral evolution as a function of sequential exponential time constants rather than actual spectra of populations (Fig. 2, *C* and *D*). In both the “PVB” and “PCB” datasets a good fit was achieved with three components, and the resulting EADS were very similar for both samples. There are slight differences in the resulting lifetimes. This is almost certainly due to the small amount of signal contamination from the PVB Pg to Pb photoconversion in the “PCB” dataset. The initial spectra, EADS1, show strong GSB and SE features, and a broad ESA, which rapidly decay in ∼2 ps to the second spectra, EADS2. These spectra still contain a strong GSB feature and broad ESA, but no obvious SE. In the final component, EADS3, which grows in from EADS2 with a lifetime of ∼10 ps, the GSB is still present, although red-shifted by ∼5 nm compared with the previous components. This apparent shift is likely to be due to the appearance of a new positive feature at ∼415 nm, corresponding to an isomerized, but still Cys-bound, intermediate (10, 11).

##### Ultrafast Transient IR Dynamics

**FIGURE 3.**
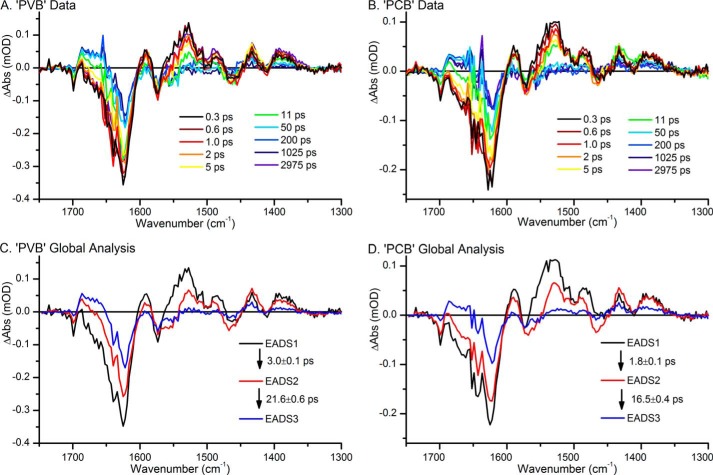
*A* and *B*, ultrafast transient IR absorption spectra, collected after excitation at 435 nm, at selected time points for “PVB” samples (*A*), where Pb states were constantly regenerated with green light, and “PCB” samples (*B*), where the Pb states were constantly regenerated with red light. *C* and *D*, global analysis of the ultrafast transient absorption data for the “PVB” samples (*C*) and “PCB” samples (*D*) which yielded three EADS that sequentially interconvert.

Ultrafast time-resolved IR spectroscopy was used to probe structural changes in the mid-infrared region of the electromagnetic spectrum after initiation of the photoisomerization reaction by 435-nm laser pulses. The difference spectra were qualitatively similar for both samples although the amplitude was reduced by approximately one third for the “PCB” sample, reflecting the lower relative concentration of PCB compared with PVB (Fig. 3, *A* and *B*). Consistent with visible ultrafast transient absorbance measurements, global analysis revealed that the data could be decomposed into three exponentially decaying species with lifetimes of τ = 3, 22, and “infinite” ps and τ = 2, 17, and infinite ps, for the “PVB” and “PCB” samples, respectively (Fig. 3, *C* and *D*).

A number of previous studies on the related phytochrome proteins allow confident assignments to be made of the features in the difference spectra shown in Fig. 3. The largest ground state bleach (negative) features occur at ∼1624 and ∼1640 cm^−1^, which correspond to C=C stretches in the C and D, and A and B rings, respectively (26, 33). The small negative feature at ∼1700 cm^−1^ is likely to correspond to the bleach of the C=O stretching mode from ring D (34, 35). The other significant bleaches at ∼1573 cm^−1^ and ∼1467 cm^−1^ are likely due to C=O and C=N stretches, respectively, both coupled to a N-H rocking motion (36). The main differences between EADS1 and EADS2 are a general loss of intensity across the whole spectral window. Features that can be attributed to the isomerized intermediate can be observed in EADS3 and include the downshift of the D-ring C=O stretch from ∼1700 to ∼1686 cm^−1^, implying a more strongly hydrogen-bonded environment in the ^15^*^E^*^-PVB′^Pb, compared with the ^15^*^Z^*^-PVB′^Pb state. Previous ultrafast IR studies have reported similar downshifts of this bond frequency upon isomerization in Cph1 (28) and two bacteriophytochromes (34), but static measurements of phytochromes have reported the opposite effect (17, 37), demonstrating the complexity of this family of photoreceptors. The C=N stretch at ∼1467 cm^−1^ may also be downshifted by the isomerization to form the small positive feature at ∼1433 cm^−1^ (36). The changes to the C=C stretches in the 1600–1650 cm^−1^ region are difficult to deconvolute, but it seems likely that the 1640 cm^−1^ feature originating from rings A and B will not change, whereas the ∼1624 cm^−1^ feature from rings C and D may shift upon photoisomerization.

##### Slow, Thermally Driven Dynamics

**FIGURE 4.**
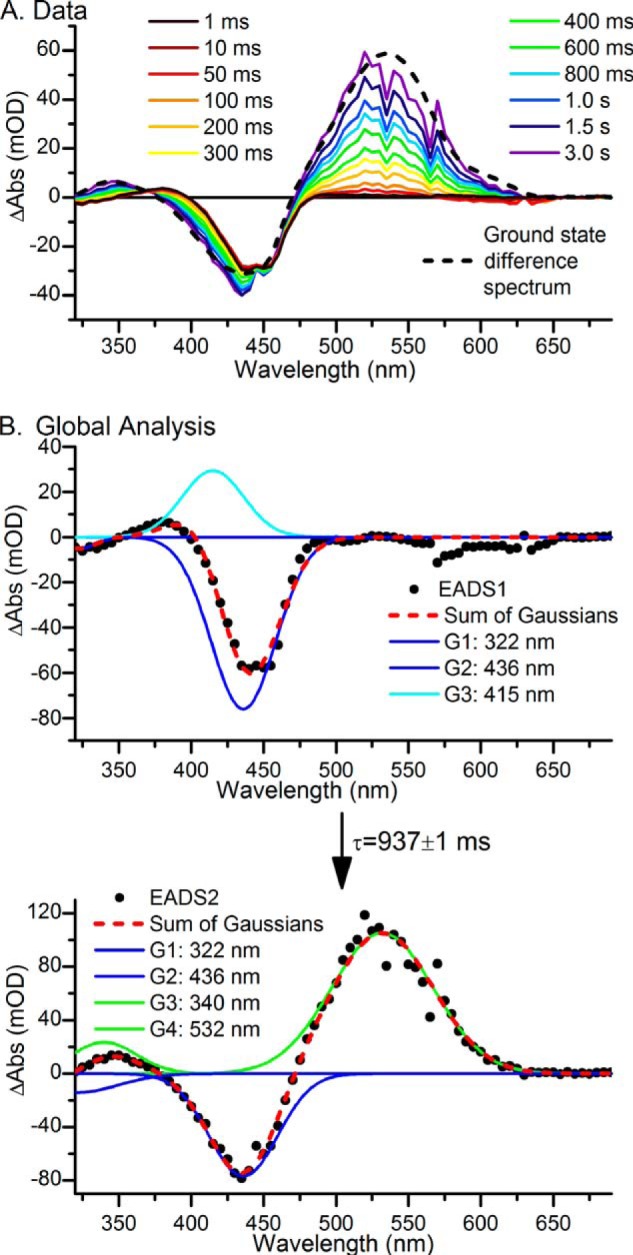
*A* and *B*, laser flash photolysis spectra after excitation at 435 nm at selected time points for a mixture of PVB and PCB Tlr0924 (*A*) and global analysis of the data showing the resulting EADS (*black dots*) fitted with a sum of Gaussian functions (*red line*) (*B*). There are obvious features originating from the ^15^*^Z^*Pb states (*blue lines*), the ^15^*^E^*Pb states (*cyan lines*), and the ^15^*^E^*^-PVB^Pg state (*green lines*). EADS1 converts to EADS2 with a lifetime of 937 ± 1 ms.

The second part of the Pb to Pg or Pr conversion involves the breakage of a thioether linkage between a cysteine residue and the methine bridge between pyrrole rings B and C (9, 10). As this is likely to proceed on much longer timescales than the isomerization, reaction intermediates were visualized on the submicrosecond to second timescale by laser and LED flash photolysis. The Pb ground state was excited with a ∼7-ns duration laser flash at 435 nm, and time-dependent absorption changes were monitored in 5-nm intervals over the 300-nm to 700-nm wavelength range. Both PVB and PCB photoproducts were returned to the Pb state between laser shots by 540-nm illumination with a cold lamp source. The time-resolved difference absorption spectra were then assembled from these data (Fig. 4*A*).

**FIGURE 5.**
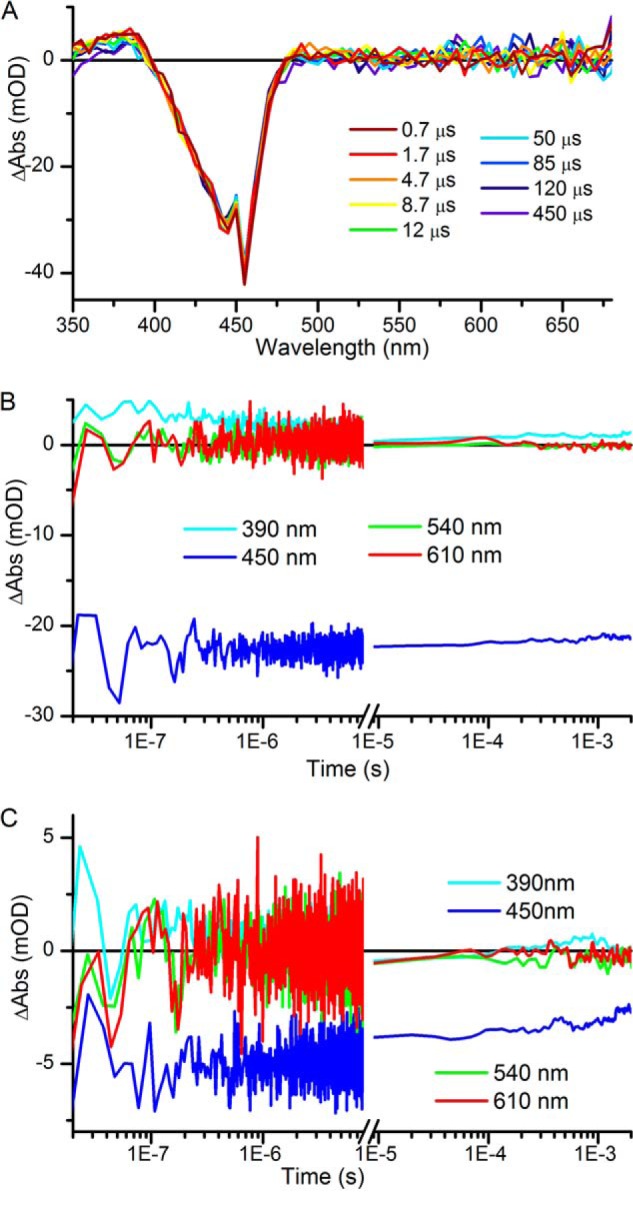
*A*, laser flash photolysis spectra at selected time points between 0. 7 and 450 μs for a mixture of PVB and PCB Tlr0924 after excitation at 435 nm. *B* and *C*, laser flash photolysis kinetics recorded in two datasets, at selected wavelength points between 20 ns and 2 ms (*B*), a mixture of PVB and PCB of Tlr0924, and PCB only Tlr0924 (*C*) after excitation at 435 nm.

After the initial photoisomerization observed in the ultrafast measurements, no further significant changes in the absorption spectra were detected between 20 ns and ∼1 ms (Fig. 5, *A* and *B*); the kinetics and difference spectra consistently show the bleach of the Pb ground states at ∼450 nm as well as a small positive feature in the 340–390-nm region. On longer timescales there is an apparent blue shift of the ground state bleach and the formation of a species at ∼345 nm, together with the appearance of the final photoproduct at ∼530 nm (Fig. 4*A*). These changes become increasingly apparent at delay times of 500 ms onward and are very similar to the static difference spectrum expected for the Pb to Pg (and Pr) photoconversion (*dotted line*). However, due to the slower reaction rate of the PCB chromophore the final spectrum measured at 3 s does lack some of the definition of the PCB photoproduct in the 600-nm region.

Global analysis of the data using a sequential model shown in Fig. 4*B* reveals an exponentially decaying component (EADS1) evolving into the final nondecaying component (EADS2) with a lifetime of 0.94 s (rate constant of 1.07 s^−1^). EADS1 can be fitted to the sum of three Gaussian functions (*red dashed line*): negative peaks at 322 and 436 nm (*blue lines*), representing the bleach of the ^15^*^Z^*Pb ground state, and a positive peak 415 nm (*cyan line*), representing the blue-shifted, ^15^*^E^*Pb, intermediate. EADS2 can be fitted to the sum of four Gaussian functions (*red dashed line*), the negative peaks at 322 and 436 nm remain, whereas the positive feature at 415 nm has been replaced by peaks at 340 and 532 nm (*green lines*), corresponding to the final ^15^*^E^*^-PVB^Pg conformation. These peak assignments are entirely consistent with previous studies on the GAF domain of Tlr0924 (5, 10, 11). The PCB population is not significantly distinct enough from the PVB population to be resolved here.

**FIGURE 6.**
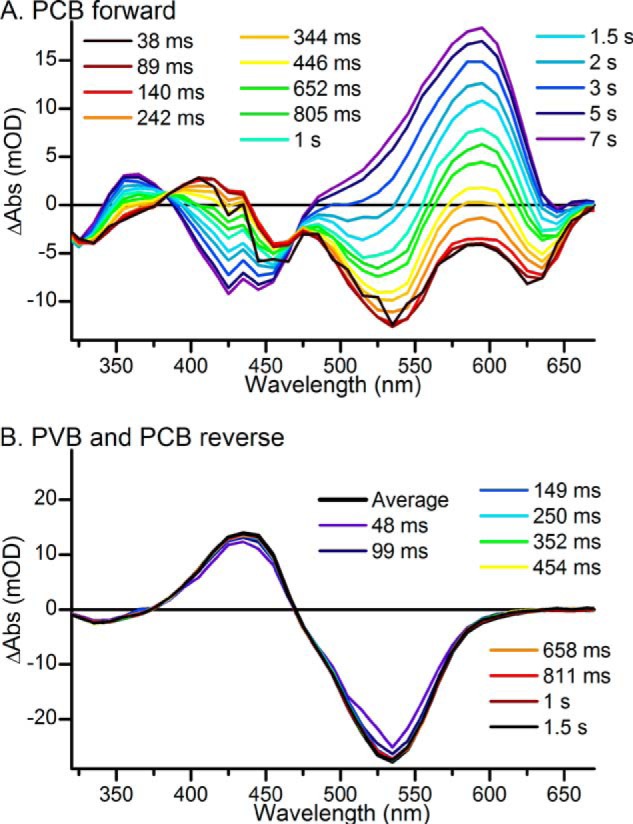
**LED flash photolysis spectra at selected time points for PCB-only forward reaction after excitation at 455 nm (*A*) and a mixture of PVB and PCB reverse photoconversion from Pg to Pb after excitation at 530 nm (*B*).**

**FIGURE 7.**
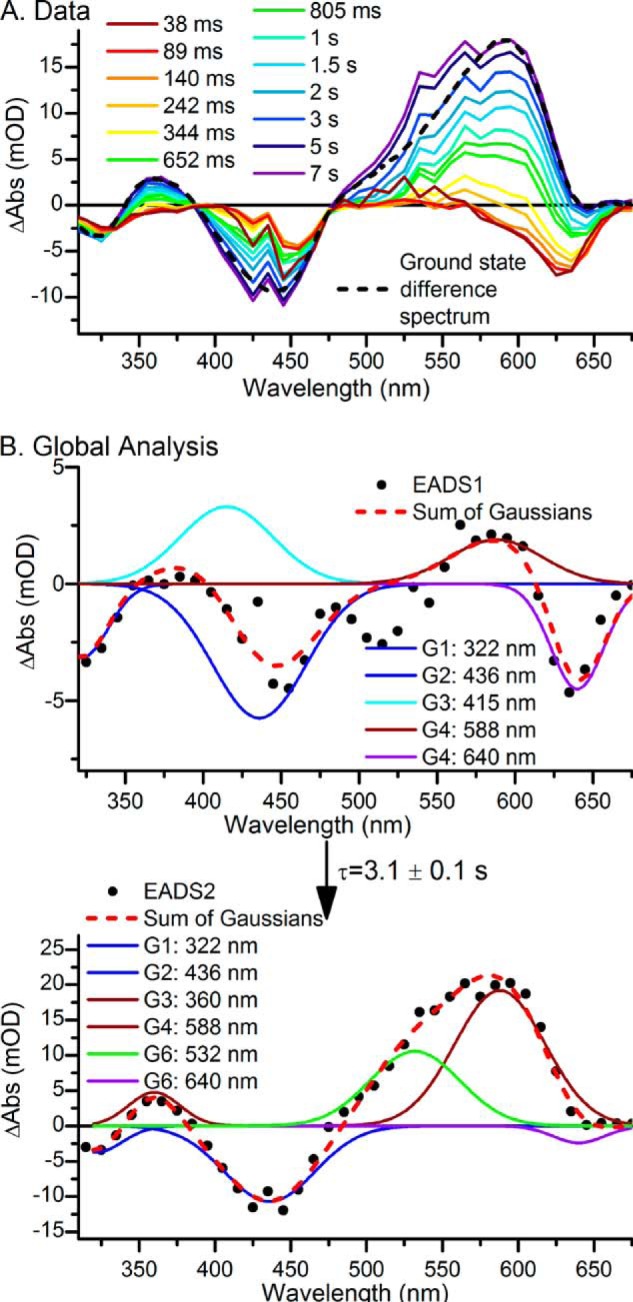
*A*, LED flash photolysis spectra at selected time points for PCB-only Tlr0924 with overlapping PVB forward and reverse reactions subtracted. *B*, global analysis of the data showing resulting EADS (*black dots*) fitted with a sum of Gaussian functions (*red line*). There are obvious features originating from the ^15^*^Z^*^-PCB′^Pb state (*blue lines*), the ^15^*^E^*^-PCB′^Pb state (*cyan lines*), the ^15^*^E^*^-PCB^Pr state (*dark red lines*), and the ^15^*^E^*^-PVB^Pg state. There is an additional feature at 640 nm (*purple line*) originating from inactive or modified protein. EADS1 converts to EADS2 with a lifetime of 3.1 ± 0.1 s.

To investigate the properties of the PCB forward photoreaction in more detail, the pure PCB′ Pb state was regenerated by illuminating the *15E* isomers (Pg and Pr) at 640 nm between each dataset collected. This converted only the PCB Pr population to the PCB′ Pb population whereas the PVB population remained in the Pg state. As with the mixture of PVB and PCB, after the initial photoisomerization observed in the ultrafast measurements there were no significant spectral changes between 20 ns and tens of ms (Fig. 5*C*). Due to the slow reaction dynamics and low concentrations of the “PCB” sample LED flash photolysis was used to monitor the dynamic processes. Samples were excited with a 10-ms, 455-nm LED flash in a UV-visible spectrophotometer to give the raw difference spectra shown in Fig. 6*A*. However, it is apparent that there were overlapping signals in the dataset, which originated from both the forward and reverse photoreactions of the PVB chromophore. Subtraction of scaled datasets collected on the same wavelength and time domains for the photoconversion of PVB from Pb to Pg (shown in Fig. 4*A*) and for the photoconversion of PVB from Pg to Pb (shown in Fig. 6*B*) resulted in the difference spectra shown in Fig. 7*A*, which should include components only from the PCB Pb to Pr photoconversion. Initial spectra show ground state bleaches at ∼325, 450, and 640 nm, and these spectra evolve over several seconds to a spectrum with a large bleach at ∼435 nm and a positive feature at ∼590 nm.

Global analysis of the data using a sequential model shown in Fig. 7*B* reveals an exponentially decaying component (EADS1) evolving into the final nondecaying component (EADS2) with a lifetime of 3.1 s (rate constant of 0.33 s^−1^). EADS1 can be fitted to the sum of five Gaussian functions (*red dashed line*): in common with the data collected from a mixture of PVB and PCB there are negative peaks at 322 and 436 nm (*blue lines*), representing the bleach of the ^15^*^Z^*Pb ground state, and a positive peak at 415 nm (*cyan line*), representing the blue-shifted ^15^*^E^*Pb intermediate. There is also a very small amount of the 588-nm product peak and an additional negative peak centered at 640 nm which correlates well with the small negative feature at ∼640 nm observed in the ultrafast measurements and may correspond to the bleach of a previously observed ground state absorption peak that was attributed to a small amount of inactive or modified protein (10). The negative feature at 640 nm remains in EADS2, which can be fitted to the sum of five Gaussian functions (red dashed line), with FWHM fixed from EADS1. In addition to the 640 nm feature the negative peaks at 322 and 436 nm remain, whereas the final ^15^*^E^*Pr photoproduct is represented by peaks at 360 and 588 nm (*dark red lines*). There is also a small amount of the PVB Pg product, represented by a positive feature at 532 nm visible, although this is likely to be due to the fallibility of the method of subtraction of the scaled datasets from the PVB forward and reverse reaction. As with the mixture of PVB and PCB, the peak assignments are entirely consistent with previous studies on the GAF domain of Tlr0924 (5, 10, 11).

##### Cryotrapping of Intermediate States

**FIGURE 8.**
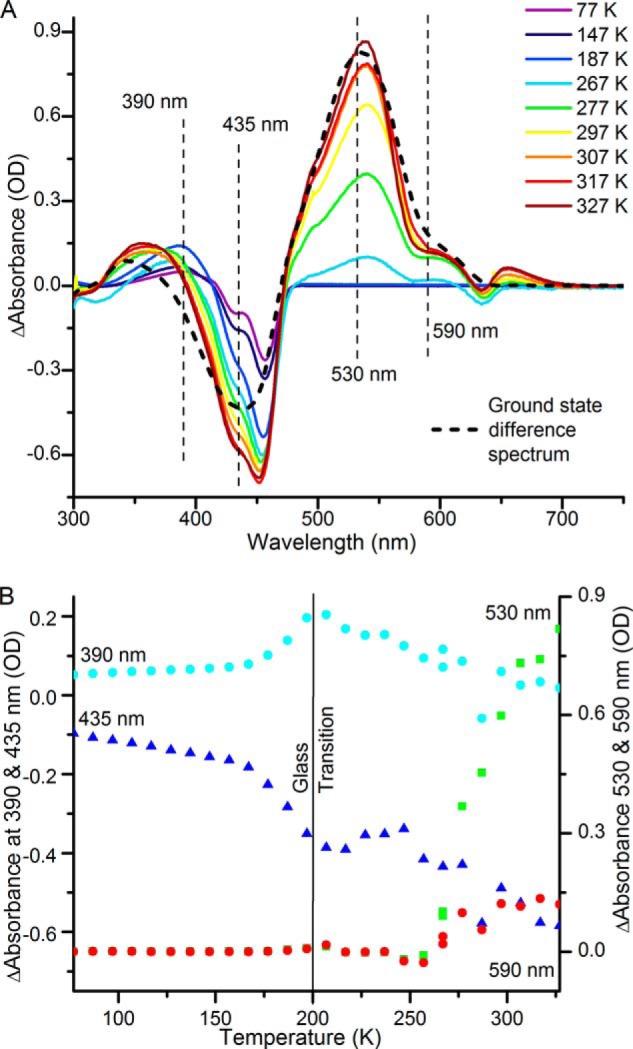
**Low temperature stabilization of reaction intermediates.** Difference spectra (*A*) and change in absorption (*B*) at 390 (*cyan*), 435 (*blue*), 535 (*green*), and 590 (*red*) nm after illumination at a range of temperatures between 77 and 327 K.

To confirm the intermediates observed by the time-resolved spectroscopy and to investigate the associated energetic or thermal barriers to these reaction steps in the Tlr0924 photocycle the photoconversion of the Pb to the Pg and Pr states was monitored by illuminating samples with blue light at at temperatures ranging from 77 to 327 K. Absorbance difference spectra were recorded at 77 K by subtraction of the initial Pb ground state absorbance spectrum (Fig. 8*A*). An initial light-dependent reaction that can be observed at temperatures below 200 K involves a bleach of the Pb ground state band at ∼450 nm and the appearance of a new absorbance band at ∼390 nm. These spectral features represent the primary ^15^*^E^*Pb photoproduct that is formed upon photoisomerization and are identical to those observed in the transient absorption measurements (see Figs. 2, 4, and 7). The temperature dependence of this photochemical step was obtained by plotting the absorbance increase at 390 nm against the temperature at which the sample was illuminated. This reveals that it has reached completion at temperatures below 200 K (Fig. 8*B*). After formation of the primary ^15^*^E^*Pb photoproduct there is a further small decrease in the main Pb absorbance band at ∼435 nm which can be observed at temperatures above 200 K, together with a shift of the small positive absorbance band at ∼390 nm to ∼345 nm (Fig. 8*A*). This is accompanied by a simultaneous increase in the absorbance peaks at ∼530 nm and ∼590 nm (Fig. 8*B*), which represent the Pg and Pr states of the protein, respectively.

## DISCUSSION

**FIGURE 9.**
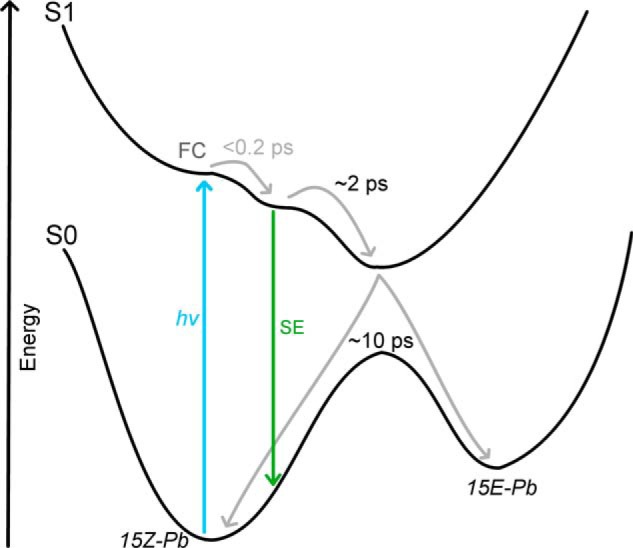
**Scheme showing suggested ground and excited state energy surfaces and the processes, which occur after photoexcitation.** *FC*, Franck-Condon region.

The combination of ultrafast visible and IR transient absorption data leads us to suggest the scheme as shown in Fig. 9 for the photoisomerization of the 15*Z* PVB′ and PCB′ species. Immediately following photoexcitation to the Franck-Condon region very fast relaxation occurs within the time resolution of our detection method (∼0.2 ps). From the Franck-Condon region an energetically excited region of the S1 potential energy surface is populated, from which SE can occur to the ground state. In ∼2 ps this state has relaxed to the energy minima of the S1 surface, and on a timescale of ∼10 ps the system relaxes to either the 15*Z* or 15*E* isomers. After this photoisomerization step the ^15^*^E^*Pb intermediate can then progress to the final ^15^*^E^*^-PVB^Pg and ^15^*^E^*^-PCB^Pr states. This scheme is less complex than, but similar to, those proposed in a previous study on the GAF domain of Tlr0924 (15) and for the corresponding isomerization process in the related Cph1 phytochrome, both of which are suggested to involve multiple intermediates on both excited state and ground state energy surfaces (26, 38, 39). Previous studies on CBCRs involved only the GAF domain, and it is possible that the full-length protein restricts the number of accessible states, resulting in the suggested simple mechanism. This would be a contrast to phytochrome systems, where even the full-length protein demonstrates multiple intermediates in the reaction pathway (2, 27, 40). The difference between the systems lies in the thioether linkage to the protein, which is lacking in phytochromes and may provide added stabilization to the CBCR reaction intermediates. It may be that after the loss of the cysteine linkage further rearrangements occur, but if these processes are fast (submicroseconds) they would not be resolved by the methods used here.

The slower, thermally driven dynamics monitored by flash photolysis measurements also present a less complex picture than that seen in many CBCR (18) and phytochrome (40) systems. It may be that there are further, spectrally identical, intermediates that we cannot distinguish using the techniques used here; however, this seems unlikely as spectral shifts between intermediates are usually >10 nm in such systems (18, 40). There appears to be only one step between the isomerization and the breaking of the Cys bond, although unlike the isomerization reaction the rate of this step appears to depend upon the properties of the chromophore. The PVB conversion completes nearly three times faster than that of PCB.

Generally, proteins undergo a dynamic transition, termed the “glass transition,” at ∼200 K, below which any large scale structural changes in the protein become frozen out (4, 41–44). As our cryotrapping measurements show, formation of the primary photoproduct can still proceed below 200 K so the photoisomerization reaction is only likely to involve structural changes within the bilin molecule itself or minor, localized protein adjustment around the bilin cofactor. This is similar to the photoisomerization reaction observed for the related Cph1 phytochrome (4). After photoisomerization both PVB and PCB reaction pathways, which involve the breakage of the thioether linkage, proceed in a single step at temperatures well above the glass transition temperature. Consequently, it is likely that there is a role for large scale protein motions that become frozen out below the glass transition temperature to eliminate the thioether linkage in Tlr0924. It is known that conformational changes and/or domain movements are crucial in the photocycle of the related Cph1 phytochrome. The formation of photoproduct occurs at much higher temperatures in Tlr0924 compared with Cph1 (4), which is likely to represent the additional energy required to break the thioether linkage.

**FIGURE 10.**
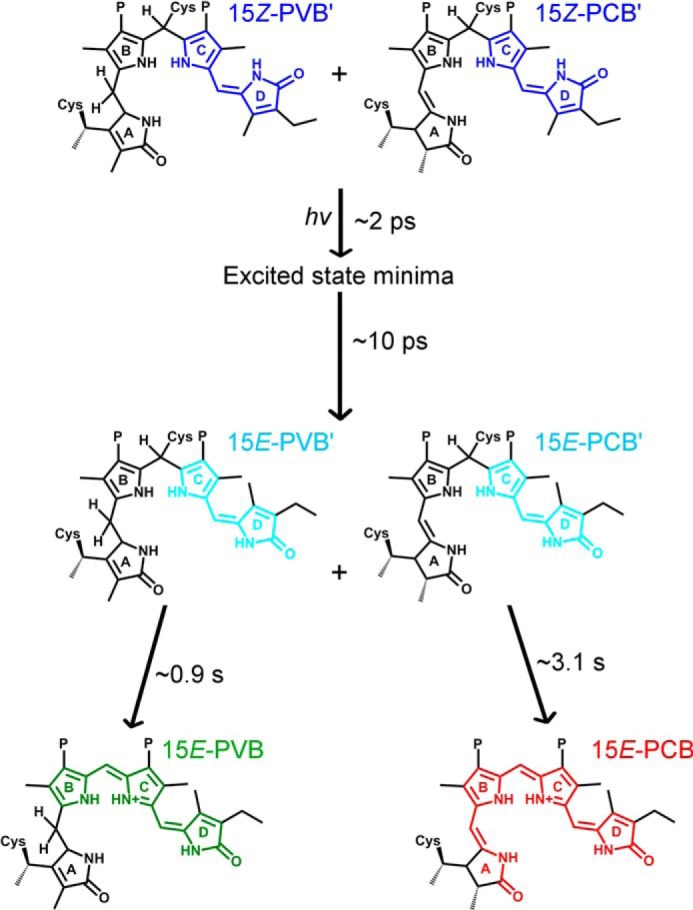
**Suggested forward reaction pathway and lifetimes for PVB and PCB in Tlr0924.** After photoexcitation both ^15^*^Z^*^-PVB′^Pb and ^15^*^Z^*^-PCB′^Pb relax to an excited state minima within 2 ps, from which isomerization to the ^15^*^E^*^-PVB′^Pb and ^15^*^E^*^-PCB′^Pb states can occur with a lifetime of ∼10 ps. At this point the photoreactions diverge with the ^15^*^E^*^-PVB^Pg and ^15^*^E^*^-PCB^Pr states being formed with a lifetimes of ∼0.9 and ∼3.1 s, respectively.

A detailed kinetic and spectroscopic analysis across a range of temperatures and timescales has provided a comprehensive understanding of the complete forward reaction dynamics during the photocycle of a full-length CBCR for the first time. By using a combination of transient spectroscopy techniques and cryotrapping measurements we propose the reaction pathway shown in Fig. 10 for the photoconversion of the Pb state to the Pg and Pr states of the protein. The PVB′ and PCB′ chromophores in Tlr0924 isomerize on a picosecond timescale, similar to that observed for other CBCR and phytochrome proteins (16, 26, 38, 39). Subsequently, the primary 15*E* PVB′ and PCB′ Pb photoproducts are converted to the final PVB Pg and PCB Pr states of the protein in slower and distinct, single-step reactions that involve the removal of a thioether linkage to a conserved cysteine residue in the protein. However, it is likely that some degree of protein conformational change is required to facilitate the removal of the thioether linkage in Tlr0924.

This proposed reaction scheme is less complex than the multiple steps (on microsecond to millisecond timescales) that follow photoisomerization in the phytochrome proteins, which are thought to involve a series of structural changes in the protein (4) and at least two metastable intermediates (17, 36). Studies of the GAF domain of CBCRs have also suggested multiple intermediates after photoisomerization in each photoconversion step (18, 20). The difference in conclusions drawn between the GAF domain only and full-length CBCRs may be explained as the full-length protein limiting the number of possible conformations for the active site, the cryotrapping measurements reported here demonstrate that protein conformational changes are involved in the loss of the second thioether linkage, giving credence to the theory that the overall protein structure affects the reaction dynamics. It is unclear whether, and to what extent, the Tlr0924 protein harbors PVB and PCB chromophores in the cell. Studies have suggested that the isomerization of PVB to PCB is a fundamental property of the GAF domain (14), and there appears to be no difference between protein coexpressed *in vitro* (as we have done) or reconstituted with the chromophore after expression (5). If the naturally occurring protein does have a heterogeneous chromophore population it may be that PCB is isomerized to PVB on the basis of faster or more efficient reaction kinetics with the remainder of the PCB population providing additional spectral coverage. In summary, this is the first analysis of the complete forward photoconversion of a full-length cyanobacteriochrome, which suggests an unprecedented slow and relatively simple reaction scheme for the thermally activated protein chemical/structural changes that follow the initial photochemical events.

## Footnotes

3 The abbreviations used are:

CBCR: cyanobacteriochrome
EADS: evolution-associated difference spectra
ESA: excited state absorption
GAF: cGMP-specific phosphodiesterase, adenylyl cyclase, and FhlA
GSB: ground state bleach
J: joules
Pb: blue-absorbing
PCB: phycocyanobilin
PCB′: C10-Cys-phycocyanobilin
Pg: green-absorbing
Pr: red-absorbing
PVB: phycoviolobilin
PVB′: C10-Cys-phycoviolobilin
SE: stimulated emission.
